# Immune indicators as predictors of cancer-related fatigue: a risk prediction model in pan-cancer patients

**DOI:** 10.3389/fragi.2025.1666116

**Published:** 2025-09-11

**Authors:** Guixin He, Ting Ge, Baohui Wang, Jianchun Yu, Wentao Li

**Affiliations:** ^1^ Department of Central Laboratory, First Teaching Hospital of Tianjin University of Traditional Chinese Medicine, Tianjin, China; ^2^ National Clinical Research Center for Chinese Medicine Acupuncture and Moxibustion, First Teaching Hospital of Tianjin University of Traditional Chinese Medicine, Tianjin, China; ^3^ Department of Graduate School, Tianjin University of Traditional Chinese Medicine, Tianjin, China; ^4^ The First Affiliated Hospital of Zhejiang Chinese Medical University (Zhejiang Provincial Hospital of Chinese Medicine), Zhejiang Chinese Medical University, Hangzhou, Zhejiang, China; ^5^ Department of Oncology, First Teaching Hospital of Tianjin University of Traditional Chinese Medicine, Tianjin, China

**Keywords:** cancer-related fatigue, logistic regression analysis, pan-cancer, risk prediction model, T-lymphocyte subsets

## Abstract

**Background:**

Cancer‐related fatigue (CRF) is a prevalent and multifactorial symptom that significantly impairs the quality of life in cancer patients. This study aimed to identify immune and clinical factors associated with CRF in a pan-cancer cohort and to develop a predictive model for CRF to inform personalized clinical management.

**Methods:**

A retrospective analysis was conducted on clinical data from 146 cancer patients admitted to the Oncology Department of the First Affiliated Hospital of Tianjin University of Traditional Chinese Medicine. The variables collected included demographic information, disease‐related data, immunological parameters, and Brief Fatigue Inventory (BFI) scores. Univariate and multivariate logistic regression analyses were used to identify independent risk factors for CRF. A predictive model was developed, and its performance was evaluated using receiver operating characteristic (ROC) curve analysis and decision curve analysis.

**Results:**

Analysis results showed that multivariate logistic regression identified increasing age, increased absolute counts (AC) of CD4+CD38−T cells, and decreased AC of CD4+CD28−T cells as independent risk factors for CRF (P < 0.05). The predictive model demonstrated moderate performance, with an area under the ROC curve (AUC) of 0.725 in the training set and 0.581 in the validation set.

**Conclusion:**

These findings suggest that chronic inflammation, potentially associated with immunosenescence and immune remodeling, may contribute to the onset of CRF. Further research is needed to validate the model in large-scale, diverse patient populations and to develop targeted interventions to alleviate fatigue and improve the quality of life in cancer patients.

## 1 Introduction

Cancer, characterized by its high incidence and mortality rates, poses a significant threat to human life and health (Siegel et al., 2023). According to the global cancer statistics for 2022, approximately 20 million individuals were newly diagnosed with cancer, and nearly 10 million succumbed to the disease (Jokhadze et al., 2024). With the continuous advancement of treatment methods, an increasing number of cancer patients are surviving and entering long-term survival periods; however, the long-term side effects and chronic health issues resulting from treatment have garnered increasing attention.

Cancer-related fatigue (CRF) is one of the most common, persistent, and challenging symptoms experienced by cancer patients and survivors (David et al., 2021). It manifests as a continuous subjective sense of physical, emotional, or cognitive fatigue that is disproportionate to recent activity levels and is not alleviated by rest or sleep, significantly impacting the patient’s daily life (Dantzer et al., 2025). Although the mechanisms underlying CRF are not yet fully elucidated, existing studies indicate that its occurrence is closely related to various factors, including demographic characteristics, clinical factors, psychological factors, and social support. For instance, research by Dickinson et al. found that factors associated with CRF include demographic variables, sleep quality, urinary issues, gastrointestinal problems, hormone-related issues, and sexual activity (Dickinson et al., 2021). Huang et al. pointed out that age, gender, low levels of physical activity, clinical staging, chemotherapy, pain, insomnia, anxiety, and depression are major risk factors for CRF (Huang et al., 2022). Furthermore, smoking, tumor recurrence, and negative coping strategies are considered risk factors for severe CRF in cervical cancer patients, while higher monthly income, exercise, and strong social support are associated with a lower risk of developing CRF (Gu et al., 2024). However, the consistency of these risk factors across different cancer types still requires further validation.

T lymphocyte subsets, which are essential for maintaining immune homeostasis, play a significant role in the inflammatory response by secreting specific cytokines (Singh et al., 2024). Studies have demonstrated that transcutaneous acupoint electrical stimulation not only significantly alleviates the fatigue experienced by patients with CRF but also enhances cell function, leading to increased absolute counts of immune cells such as CD3^+^T, CD4^+^T, and CD8^+^T cells (Shu et al., 2022). Recent studies indicate that the imbalance in T cell subpopulation ratios correlates with the onset of CRF. The Spleen-invigorating and Blood-regulating Decoction may exert its anti-inflammatory effects by inhibiting the expression of Ki-67, which could improve the Th1/Th2 balance and alleviate fatigue in mice with liver cancer (Jiayi et al., 2024). Furthermore, the inflammatory response is regarded as a major driving factor of the gut-brain axis in CRF (Xiao et al., 2021). Various inflammatory cytokines (including IL-4, IL-6, IL-8, IL-10, and IL-1β) and tumor necrosis factor-alpha (TNF-α) are significantly associated with CRF (Song et al., 2024). However, the role of T lymphocyte subsets in predicting CRF has yet to be systematically evaluated across different cancers.

Currently, research on CRF prediction models is relatively limited, predominantly focusing on single cancer types. Comparative studies across different cancer types are scarce, and the exploration of immune-related influencing factors remains insufficient. The primary risk factors affecting CRF in cancer patients have not been identified, thereby restricting the universality and accuracy of CRF prediction models. Consequently, identifying the key factors influencing CRF in pan-cancer patients and developing corresponding risk prediction models is of significant clinical importance. This study employs a retrospective analysis method, incorporating potential risk factors such as age, gender, marital status, family history, smoking status, alcohol consumption, clinical staging, surgical conditions, pathological status, and absolute counts of T lymphocyte subpopulations. It analyzes the impact of these factors on CRF in patients with various cancers, including breast, lung, liver, colorectal, prostate, gastric, pancreatic, and esophageal cancers. Utilizing univariate and multivariate logistic regression analysis, a risk prediction model for CRF was constructed, and the model’s predictive performance and calibration were evaluated using the receiver operating characteristic (ROC) curve. The predictive factors identified in this model are easily accessible in clinical settings, facilitating the timely identification of high-risk patients and providing new insights for formulating effective intervention strategies to mitigate CRF in cancer patients.

## 2 Materials and methods

### 2.1 Research objects

**FIGURE 1:**
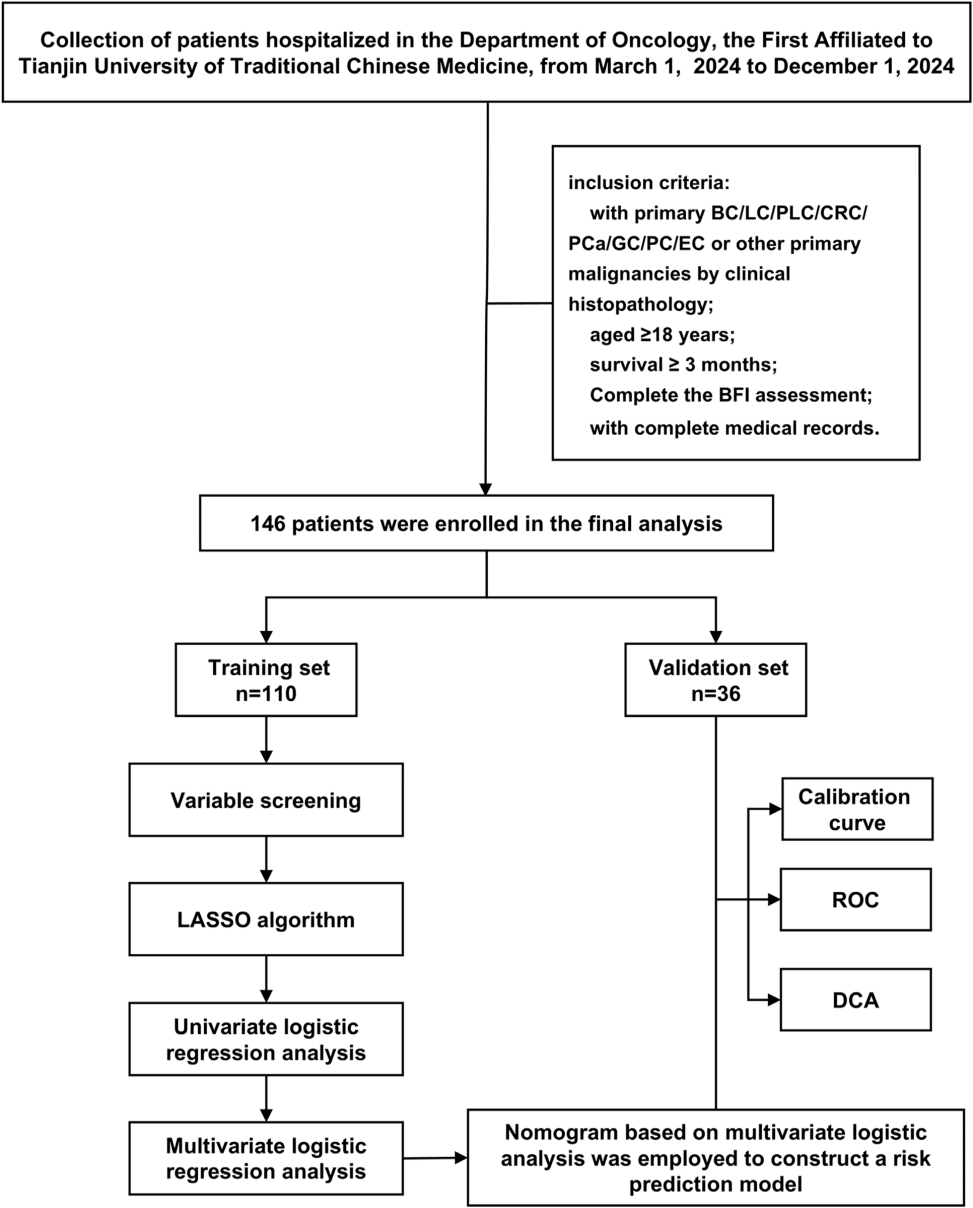
Flow chart of the study.

This study conducted a retrospective analysis of 146 cancer patients who met the inclusion and exclusion criteria and received treatment at the Oncology Department of the First Affiliated Hospital of Tianjin University of Traditional Chinese Medicine from March 2024 to December 2024. The patient screening process and study design are illustrated in Figure 1. This study has been approved by the Ethics Committee of our hospital (Approval No: TYLL2025 [K]006, 17 April 2025). Given the retrospective nature of this study, we waived the requirement for obtaining written informed consent.

### 2.2 Inclusion criteria (those who meet the following five conditions are included)

The inclusion criteria for this study are as follows: (1) Inpatients diagnosed with primary breast cancer (BC), lung cancer (LC), primary liver cancer (PLC), colorectal cancer (CRC), prostate cancer (PCa), gastric cancer (GC), pancreatic cancer (PC), or esophageal cancer (EC), or other malignancies through clinical histopathology to have any of the aforementioned cancers; (2) possession of complete medical records; (3) aged ≥18 years; (4) survival ≥3 months; and (5) completion of the Brief Fatigue Inventory (BFI) assessment at the study time point, with fatigue severity categorized according to BFI cut-off values.

Note: For the purpose of this study, fatigue status was determined exclusively based on the BFI score obtained at the assessment time point, irrespective of any prior CRF diagnosis documented in the medical records.

### 2.3 Exclusion criteria (excluding those who meet any of the following criteria)

(1) Patients with secondary tumors or concomitant tumors; (2) Patients with severe complications, including cardiac, hepatic, renal, or hematological issues; (3) Patients with a history of psychiatric disorders or cognitive impairments.

### 2.4 Data collection

#### 2.4.1 Demographic information

Gender (Male/Female), Age (years), Marital status (Single/Married/Divorced/Widowed), Family history (Yes/No), Smoking history (Yes/No), Drinking history (Yes/No).

#### 2.4.2 Clinical information

Clinical Stage (0/I/II/III/IV), Surgical history (Yes/No), Treatment Methods (Radiotherapy/Chemotherapy/Immunotherapy/Targeted therapy).

#### 2.4.3 Immunological indicators

The absolute counts (AC) of the following immune cells were collected: CD3+T cells, CD4+T cells, CD8+T cells, CD4^+^CD38+T cells, CD4^+^CD38-T cells, CD4^+^CD28+T cells, CD4^+^CD28-T cells, CD8^+^CD38+T cells, CD8^+^CD38-T cells, CD8^+^CD28+T cells, CD8^+^CD28-T cells, Treg, CD4^+^ naïve T cells (Tn), and CD8+Tn cells.

### 2.5 BFI score

BFI comprises nine items, organized into two sections. The first three items evaluate the current fatigue level, the worst fatigue experienced in the past 24 h, and the usual fatigue level. The mean of these three scores constitutes the fatigue intensity score. The subsequent six items assess the impact of fatigue on various aspects, including general activities, mood, actions, daily work, relationships, and enjoyment of life, with their mean representing the fatigue impact score. The total BFI score is derived from the average of all nine items, ranging from 0 to 10; a higher score indicates a greater level of fatigue. Fatigue levels are categorized based on the total score: no fatigue (0 points), mild fatigue (1–3 points), moderate fatigue (4–6 points), and severe fatigue (7–10 points) (Mendoza et al., 1999). In the binary logistic regression analysis of fatigue predictors, fatigue severity was classified as a binary variable (no/mild fatigue: <4 points; moderate/severe fatigue: ≥4 points) to provide a clearer depiction of the fatigue status of the study subjects (Franz et al., 2019). Notably, this classification was based solely on the BFI score at the time of assessment, and patients with a documented CRF diagnosis but a BFI score of 0 were included in the non-fatigue/mild-fatigue group.

### 2.6 Flow cytometry instruments and antibodies

Flow cytometric analysis was performed using a 10-color BD FACS Canto Plus flow cytometer (BD Biosciences, United States; serial number U6573380-00541). Peripheral blood lymphocyte subsets, including CD3^+^T cells, CD4^+^T cells, and CD8^+^T cells, were identified based on the expression of specific surface markers. Within the CD4^+^T cell population, further subpopulations were classified as activated T cells, naïve T cells (Tn), and regulatory T cells (Treg), whereas within the CD8^+^T cell population, further subpopulations were classified as activated T cells and Tn. All monoclonal antibodies used for immunophenotyping were purchased from BD Biosciences (United States) and included the following: V500-C Mouse Anti-Human CD45 (Cat. No. 662912), APC-H7 Mouse Anti-Human CD3 (Cat. No. 663490), PerCP-Cy5.5 Mouse Anti-Human CD3 (Cat. No. 652831), PE-Cy7 Mouse Anti-Human CD4 (Cat. No. 663493), FITC Mouse Anti-Human CD4 (Cat. No. 340133), PerCP-Cy5.5 Mouse Anti-Human CD8 (Cat. No. 665335), APC Mouse Anti-Human CD38 (Cat. No. 345807), PE Mouse Anti-Human CD28 (Cat. No. 662797), PE Mouse Anti-Human CD127 (Cat. No. P010034-B), APC Mouse Anti-Human CD25 (Cat. No. 662525), FITC Mouse Anti-Human CD45RA (Cat. No. 662840), BV421 Mouse Anti-Human CD62L (Cat. No. 563862), PerCP-Cy5.5 Mouse Anti-Human CD95 (Cat. No. 561655), and PE Mouse Anti-Human CD45RO (Cat. No. 663530). Additional reagents included BD Trucount™ Absolute Count Tubes (Cat. No. 340334) and BD Multitest™ IMK Lysing Solution 10× (Cat. No. 340503).

### 2.7 Lymphocyte subsets enumeration and phenotyping

**FIGURE 2:**
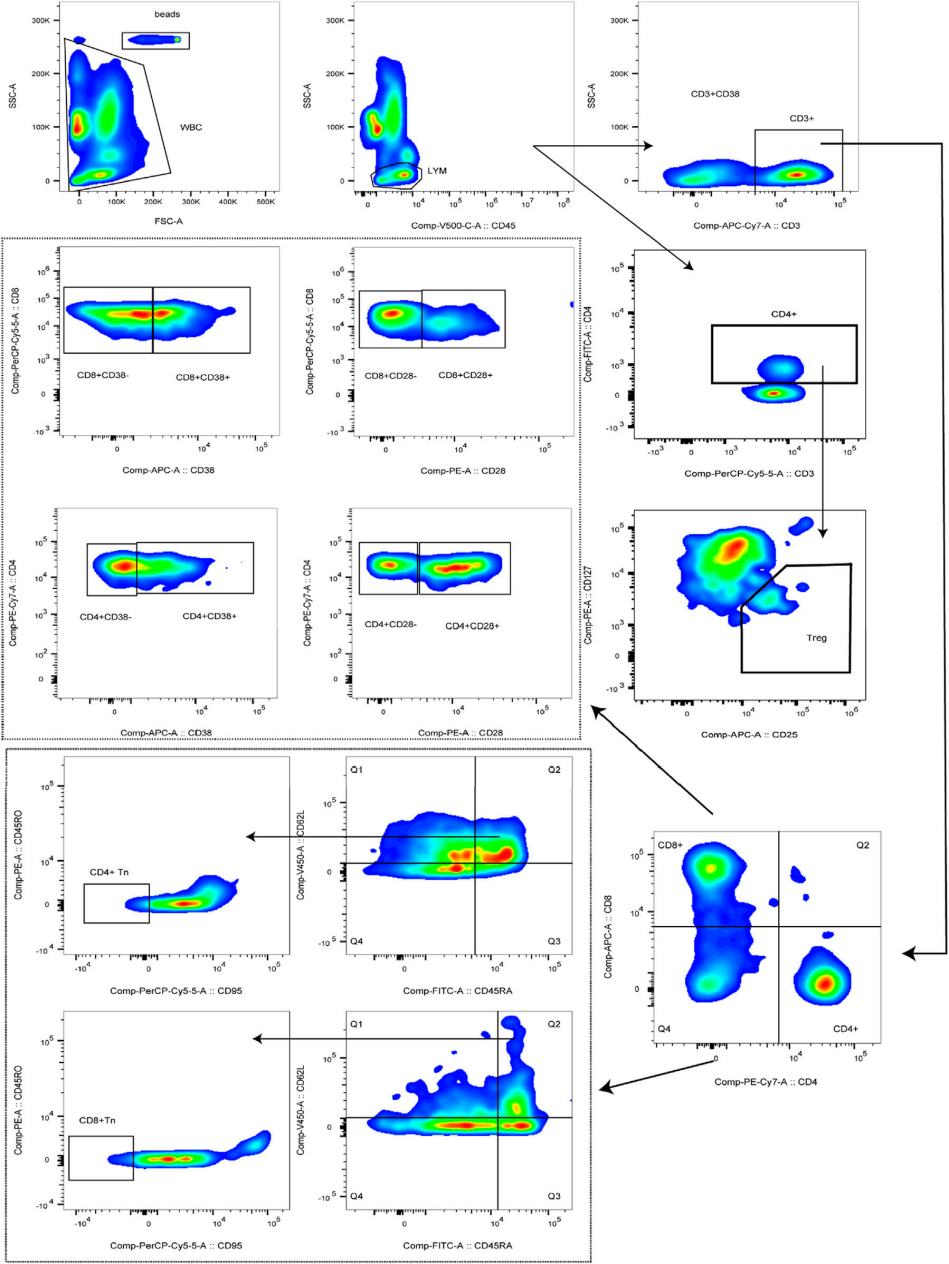
Gating strategies for Peripheral blood lymphocyte subsets.

Peripheral blood (2 mL) was collected from hospitalized patients into EDTA anticoagulant tubes, gently mixed, and immediately transported to the laboratory for flow cytometric analysis. BD Trucount™ Absolute Count tubes were prepared according to the number of samples, and each tube was labeled with a unique identifier. For Tn cell detection, 5μL each of anti-CD45, anti-CD3, anti-CD4, anti-CD8, anti-CD62L, and anti-CD95 antibodies, together with 20μL each of anti-CD45RA and anti-CD45RO antibodies, were added to the corresponding tubes. For activated T cell detection, 5μL each of anti-CD45, anti-CD3, and anti-CD4 antibodies, together with 20μL each of anti-CD8, anti-CD38, and anti-CD28 antibodies, were added. For Treg cell detection, 5μL each of anti-CD45 and anti-CD25 antibodies, together with 20μL each of anti-CD3, anti-CD4, and anti-CD127 antibodies, were added. Subsequently, 50μL of well-mixed anticoagulated whole blood was dispensed into each tube, gently mixed, and incubated in the dark at room temperature for 15 min. Thereafter, 450μL of 1×BD Multitest™ IMK Lysing Solution was added, the tubes were gently mixed, and incubation was continued under the same conditions for an additional 15min. Flow cytometric acquisition was performed using the flow cytometer, and absolute cell counts were calculated according to the following formula: Cells/μL = (acquired cells * total beads)/(acquired beads * sample volume). Cell populations were defined as follows: T cells were identified as CD3^+^, CD4^+^T cells as CD3^+^CD4^+^, and CD8^+^T cells as CD3^+^CD8^+^. Activated T cells were identified as CD3^+^CD4^+^CD28^+^, CD3^+^CD4^+^CD28^−^, CD3^+^CD4^+^CD38^+^, CD3^+^CD4^+^CD38^−^, CD3^+^CD8^+^CD28^+^, CD3^+^CD8^+^CD28^−^, CD3^+^CD8^+^CD38^−^, or CD3^+^CD8^+^CD38^−^. Tn cells were defined as CD45RA^+^CD62L^+^CD95^−^CD45RO^−,^ and Treg cells were defined as CD4^+^CD25^+^CD127^-^. The complete gating strategy used for the identification of these subsets is presented in Figure 2.

### 2.8 Statistics

This study employed SPSS version 25.0 and R version 4.3.2 for statistical analyses. Continuous data that followed a normal distribution were expressed as ( $\bar{x}$ ± s), while inter-group comparisons were conducted using the independent samples t-test. Non-normally distributed data were represented as M (*P*25, *P*75), with the Mann-Whitney *U* rank-sum test applied for inter-group comparisons. Categorical variables were reported as frequencies and percentages, with the Chi-square test applied to evaluate differences between groups. To select variables for model construction, we applied the least absolute shrinkage and selection operator (LASSO) regression using the “glmnet” package in R. LASSO regression was selected because it is particularly suitable for high-dimensional datasets, effectively addresses multicollinearity among predictors, and simultaneously performs variable selection and regularization, thereby enhancing model stability and predictive performance. All categorical variables were converted into dummy variables before analysis. Ten-fold cross-validation was conducted to determine the optimal penalty parameter, and variables with non-zero coefficients at the λ 1se were considered significant and subsequently included in both univariate and multivariate logistic regression analyses to identify independent predictors of CRF. A risk prediction model was then developed using the “rms” package, and the ROC curve was generated with the “pROC” package to compute the area under the curve (AUC), which assesses the model’s discriminative ability. The model’s calibration was evaluated through calibration curves, with a strong overlap between the calibration curve and the ideal curve indicating a good model fit. Finally, decision curve analysis (DCA) was conducted using the “rmda” package to evaluate the clinical utility of the model across various risk thresholds.

## 3 Results

### 3.1 Comparison of baseline data and analysis of factors affecting CRF in cancer patients

This study included a total of 146 cancer patients. Based on a risk ratio of 4:1, the training set included 30 randomly selected samples, while the validation set included 85 samples. No statistically significant differences were observed in the baseline data between the model training set and the validation set (*P* > 0.05), as illustrated in Table 1. Among the total sample, according to the BFI scoring criteria, the rate of no or mild fatigue was 36.3% (53/146), whereas the rate of moderate to severe fatigue was 63.7% (93/146). The incidence of moderate to severe fatigue in the model training set was recorded at 65.45%. The distribution of cancer types is detailed in Table 2, with no statistically significant difference in fatigue incidence among cancer types (*P* = 0.611). Factors influencing the occurrence of CRF in patients included age, CD4^+^CD38^−^T AC, and CD4^+^CD28^−^T AC (*P* < 0.05), as shown in Supplementary Table S1.

**TABLE 1:** Patient demographics and baseline characteristics.

Characteristic	Cohort		*p*-value^a^
	Training set, n = 110^b^	Validation set, n = 36^b^	
Age			0.126
Mean ± SD	66 ± 10	63 ± 11	
Gender			0.236
Male	49 (44.5%)	12 (33.3%)	
Female	61 (55.5%)	24 (66.7%)	
Marital status			0.333
Single	3 (2.7%)	0 (0.0%)	
Married	97 (88.2%)	31 (86.1%)	
Divorced	1 (0.9%)	2 (5.6%)	
Widowed	9 (8.2%)	3 (8.3%)	
Clinical stages			0.157
0	7 (6.4%)	3 (8.3%)	
Ⅰ	23 (20.9%)	5 (13.9%)	
Ⅱ	18 (16.4%)	3 (8.3%)	
Ⅲ	28 (25.5%)	17 (47.2%)	
Ⅳ	34 (30.9%)	8 (22.2%)	
Family history			0.353
No	87 (79.1%)	31 (86.1%)	
Yes	23 (20.9%)	5 (13.9%)	
Smoking history			0.101
No	69 (62.7%)	17 (47.2%)	
Yes	41 (37.3%)	19 (52.8%)	
Drinking history			0.942
No	88 (80.0%)	29 (80.6%)	
Yes	22 (20.0%)	7 (19.4%)	
Surgical history			0.601
No	68 (61.8%)	24 (66.7%)	
Yes	42 (38.2%)	12 (33.3%)	
Radiotherapy			0.328
No	88 (80.0%)	26 (72.2%)	
Yes	22 (20.0%)	10 (27.8%)	
Chemotherapy			0.058
No	47 (42.7%)	9 (25.0%)	
Yes	63 (57.3%)	27 (75.0%)	
Immunotherapy			0.122
No	82 (74.5%)	22 (61.1%)	
Yes	28 (25.5%)	14 (38.9%)	
Targeted therapy			0.339
No	65 (59.1%)	18 (50.0%)	
Yes	45 (40.9%)	18 (50.0%)	
CD3^+^T AC (cells/μL)			0.844
Mean ± SD	671 ± 362	657 ± 340	
CD4^+^T AC (cells/μL)			0.307
Mean ± SD	367 ± 188	330 ± 187	
CD8^+^T AC (cells/μL)			0.594
Mean ± SD	228 ± 157	247 ± 191	
CD4^+^CD38^+^T AC (cells/μL)			0.549
Mean ± SD	47 ± 49	43 ± 33	
CD4^+^CD38^-^T AC (cells/μL)			0.869
Mean ± SD	76 ± 59	77 ± 41	
CD4^+^CD28^+^T AC (cells/μL)			0.139
Mean ± SD	87 ± 66	74 ± 37	
CD4^+^CD28^-^T AC (cells/μL)			0.534
Mean ± SD	14 ± 7	13 ± 5	
CD8^+^CD38^+^T AC (cells/μL)			0.603
Mean ± SD	41 ± 31	38 ± 29	
CD8^+^CD38^-^T AC (cells/μL)			0.744
Mean ± SD	103 ± 51	101 ± 39	
CD8^+^CD28^+^T AC (cells/μL)			0.142
Mean ± SD	141 ± 108	120 ± 63	
CD8^+^CD28^-^T AC (cells/μL)			0.803
Mean ± SD	22 ± 9	22 ± 9	
Treg AC (cells/μL)			0.247
Mean ± SD	28 ± 15	31 ± 17	
CD4^+^Tn AC (cells/μL)			0.193
Mean ± SD	15 ± 24	11 ± 11	
CD8^+^Tn AC (cells/μL)			0.094
Mean ± SD	7 ± 10	11 ± 13	

^b^ n (%).

^a^ Welch Two-Sample t-test; Pearson’s Chi-squared test; Fisher’s exact test.

**TABLE 2:** Distribution of cancer types and incidence of fatigue across cancer types.

Cancer type	n (%)	No/Mild fatigue, n (%)	Moderate/Severe fatigue, n (%)	*P*-value^a^
Breast	10 (6.8%)	5 (50.0%)	5 (50.0%)	
Colorectal	14 (9.6%)	3 (21.4%)	11 (78.6%)	
Esophageal	8 (5.5%)	3 (37.5%)	5 (62.5%)	
Gastric	8 (5.5%)	2 (25.0%)	6 (75.0%)	
Liver	4 (2.7%)	2 (50.0%)	2 (50.0%)	
Lung	67 (45.9%)	28 (41.8%)	39 (58.2%)	
Pancreatic	9 (6.2%)	1 (11.1%)	8 (88.9%)	
Prostate	7 (4.8%)	2 (28.6%)	5 (71.4%)	
Other	19 (13.0%)	7 (36.8%)	12 (63.2%)	
Total	**146**	**53 (36.3%)**	**93 (63.7%)**	**0.611**

^a^ P-value calculated using Fisher’s Exact Test (two-sided) due to >20% of cells with expected count <5. Fatigue severity classified according to BFI, total score: No/mild fatigue (<4), Moderate/severe fatigue (≥4).

### 3.2 Screening of risk variables for CRF in pan-cancer patients

**FIGURE 3:**
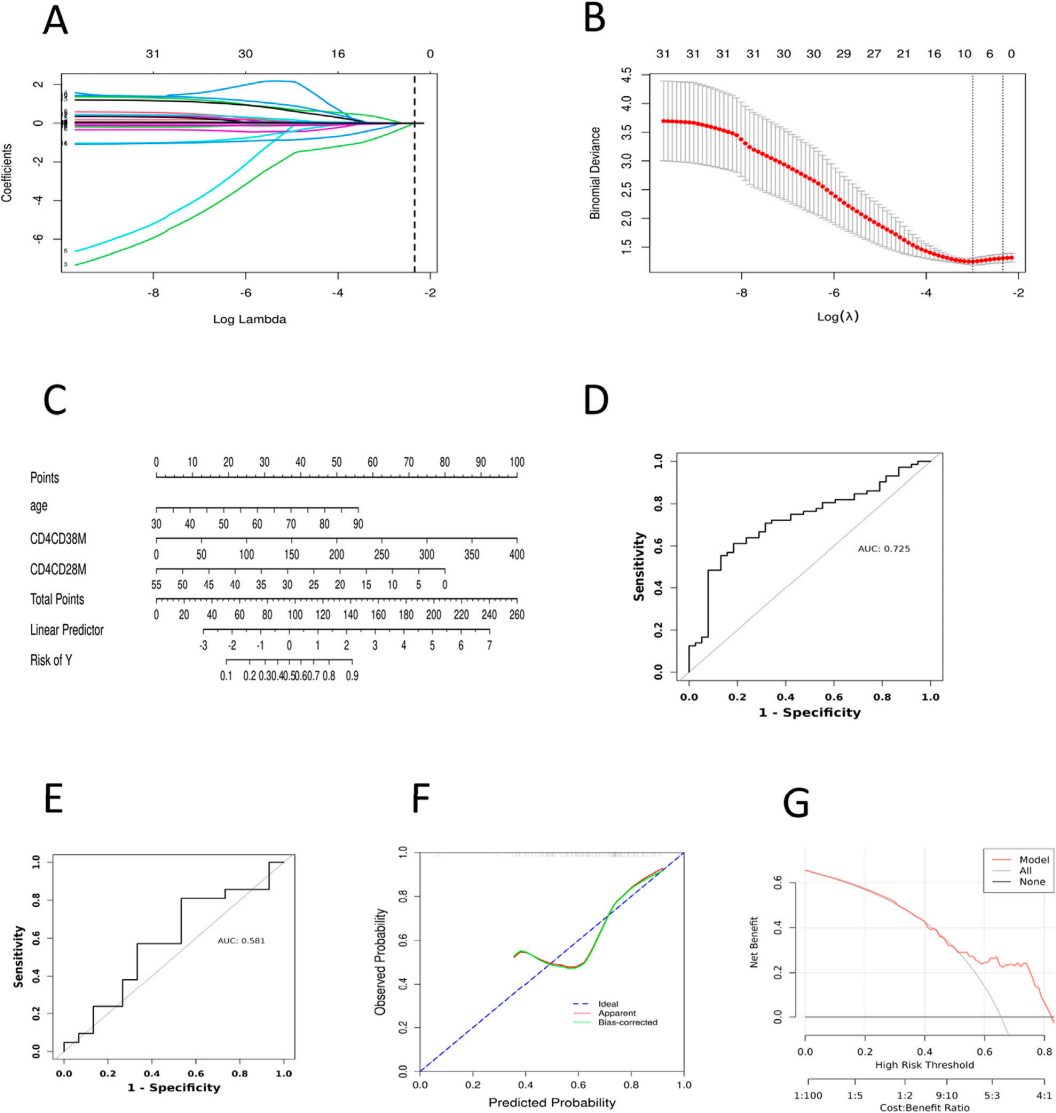
Construction and Validation of the CRF Prediction Model in Pan-Cancer Patients. **(A)** Lasso regression analysis of the coefficients. **(B)** Cross-validation for tuning the parameter selection in LASSO regression. **(C)** Nomogram prediction for the risk of CRF in patients with pan-cancer. **(D)** ROC curve of the CRF prediction model for pan-cancer patients in the model training set. **(E)** ROC curve of the CRF prediction model for pan-cancer patients in the model validation set. **(F)** Calibration curve of the training set. **(G)** DCA curve of the training set. ROC, Subjects’ Functional Curve; AUC, Area Under ROC Curve; CRF, Cancer-related Fatigue. CD4CD28M, CD4^+^CD28^−^T AC; CD4CD38M, CD4^+^CD38^−^T AC.

This study investigated CRF as the dependent variable in patients with pan-cancer, categorizing it into two levels: 0 for absent or mild fatigue and 1 for moderate or severe fatigue. Several indicators, including patients’ demographic characteristics, clinical information, and absolute counts of T lymphocyte subpopulations, were used as independent variables. For variable selection, the LASSO regression was applied. All categorical variables were transformed into dummy variables, and 10-fold cross-validation was performed to determine the optimal lambda value, with the lambda 1se set at 3 (Figures 3A,B). The LASSO regression coefficients are presented in Supplementary Table S2. The analysis identified three significant predictors: age, CD4^+^CD38^−^T AC, and CD4^+^CD28^−^T AC. These variables were subsequently entered into both univariate and multivariate logistic regression analyses. In the univariate logistic regression (Table 3), age (OR = 1.05, 95% CI: 1.01–1.09, *P* = 0.022) and CD4^+^CD38^−^T AC (OR = 1.01, 95% CI: 1.00–1.02, *P* = 0.018) were positively associated with CRF, whereas CD4^+^CD28^−^T AC (OR = 0.93, 95% CI: 0.87–0.99, *P* = 0.019) was inversely associated, suggesting a potential protective effect. In the multivariate logistic regression (Table 4), all three predictors retained statistically significant associations with CRF after adjustment for the other variables: age (OR = 1.05, 95% CI: 1.00–1.09, *P* = 0.041), CD4^+^CD38^−^T AC (OR = 1.01, 95% CI: 1.00–1.02, *P* = 0.040), and CD4^+^CD28^−^ T AC (OR = 0.93, 95% CI: 0.87–1.00, *P* = 0.049). These findings indicate that both immunological parameters and age are independent predictors of CRF in patients with various cancers, underscoring the potential role of T-cell immunophenotypes in CRF pathogenesis and offering insights for targeted risk assessment and intervention strategies.

**TABLE 3:** Results of univariate logistic regression.

Characteristic	N	Event N	OR^a^	95% CI^a^	*p*-value
Age	110	72	1.05	1.01, 1.09	0.022
CD4^+^CD38^−^T AC	110	72	1.01	1.00, 1.02	0.018
CD4^+^CD28^−^T AC	110	72	0.93	0.87, 0.99	0.019

^a^ OR, odds ratio; CI, confidence interval.

**TABLE 4:** Results of multivariate logistic regression.

Characteristic	N	Event N	OR^a^	95% CI^a^	*p*-value
Age	110	72	1.05	1.00, 1.09	0.041
CD4^+^CD38^−^T AC	110	72	1.01	1.00, 1.02	0.040
CD4^+^CD28^−^T AC	110	72	0.93	0.87, 1.00	0.049

^a^ OR, odds ratio; CI, confidence interval.

### 3.3 Development of a risk prediction model for CRF in pan-cancer patients

Based on the findings of the multifactorial logistic regression analysis, we employed the ‘rms’ package in R to visually analyze three independent predictive factors: age, CD4^+^CD38^−^T AC and CD4^+^CD28^−^T AC cells, thus constructing a nomogram model to predict the risk of CRF in patients with pan-cancer (Figure 3C). In this nomogram, the total score is computed by summing the corresponding scores of each variable, ranging from 0 to 100. A higher total score signifies an increased risk of CRF for the patient. The findings indicate that among patients with pan-cancer, older age, a higher absolute count of CD4^+^CD38^−^T cells in peripheral blood, and a lower absolute count of CD4^+^CD28^−^T cells are associated with a heightened risk of developing CRF.

### 3.4 Evaluation of the predictive efficacy of the CRF risk prediction model in pan-cancer patients

To assess the predictive capability of the nomogram model, we plotted the ROC curves for both the model training and validation sets (Figures 3D,E), which revealed AUC values of 0.725 and 0.581, respectively. Additionally, we plotted the ROC curves for age, CD4^+^CD38^−^T AC, and CD4^+^CD28^−^T AC, yielding AUC values of 0.622, 0.628, and 0.641 (Supplementary Figure S1), respectively. These results indicate that the predictive ability of individual variables is relatively limited, whereas comprehensive modeling demonstrates a higher discriminative power. The low degree of overlap between the calibration curve and the ideal curve suggests a certain deviation between the model’s predictions and the actual occurrence of risk (Figure 3F; Supplementary Figure S2). This deviation may arise from issues such as model overfitting or underfitting, insufficient feature selection, or the inherent imbalance in the data. Furthermore, we assessed the clinical validity of the model using the DCA curve. The DCA curves exhibited minimal variation across different threshold probability ranges, with threshold probabilities concentrated around 50% (Figure 3G; Supplementary Figure S3). These findings suggest that the net benefit of clinical intervention for patients within this range is limited, and the clinical utility of the model remains relatively stable. Nevertheless, this result still demonstrates that the nomogram model possesses some risk prediction capability and preliminary potential for clinical application in pan-cancer patients. However, further optimization is required in terms of variable selection, model optimization, and risk threshold setting to enhance the model’s clinical decision support capabilities.

## 4 Discussion

CRF is a common adverse reaction experienced by cancer patients, significantly impacting their treatment outcomes and quality of life. In recent years, an increasing number of studies have indicated that the occurrence of CRF is not only related to psychosocial factors (Brownstein et al., 2022) but also involves multiple aspects, including immunity, inflammation, metabolism, and neuroendocrine systems (Xin et al., 2023). Most existing CRF prediction models have relied primarily on demographic, clinical, and psychosocial variables—such as age, disease stage, treatment modality, anemia, sleep disturbance, and psychological distress—achieving a certain level of predictive performance in specific cancer populations (Gu et al., 2024; Zhang et al., 2024). However, these models rarely incorporate objective immune parameters and are seldom validated across multiple cancer types. This study, through retrospective analysis, identified that age, CD4^+^CD38^−^T AC, and CD4^+^CD28^−^T AC are significantly associated with CRF, thereby providing a reference for further understanding the biological mechanisms of CRF and for the development of its predictive models.

Age, as an independent risk factor influencing CRF, has been widely confirmed across various types of cancer. Elderly patients are more susceptible to CRF than their younger counterparts due to factors such as declining immune function (Lewis et al., 2022), diminished physical capabilities (Arzu and Özlem, 2021), and insufficient psychosocial support (Tacara et al., 2022). Furthermore, older adults may experience cognitive decline or communication barriers, which can affect the accuracy of assessments and further complicate the evaluation of CRF (Pergolotti et al., 2020). Although aerobic exercise and strength training have been validated as effective methods for alleviating CRF (Ee et al., 2024), elderly patients may experience limited benefits due to decreased muscle mass and lower levels of physical activity. Therefore, clinical practice for elderly cancer patients should adopt a comprehensive intervention strategy that integrates exercise interventions, psychological support, and immunomodulation, among other measures, to more effectively alleviate CRF.

This study further revealed that alterations in CD4^+^ T cell subsets play an important role in the development of CRF. Specifically, increased absolute counts of CD4^+^CD38^−^T cells and decreased absolute counts of CD4^+^CD28^−^ T cells were both significantly associated with CRF, suggesting that chronic inflammation related to immunosenescence and immune remodeling may contribute to its pathogenesis. CD4^+^ T cells are a critical component of the immune system and play essential roles in immune regulation and anti-tumor responses (Speiser et al., 2023). They can directly kill tumor cells or differentiate into Th1 cells, exerting cytotoxic effects through the secretion of perforin or granzyme-dependent mechanisms, or by eliminating tumors via the Fas/FasL system. Additionally, they can secrete cytokines such as IL-1 and IFN-γ, which activate M1 macrophages, thereby facilitating the clearance of tumor cells (Dipalma and Blattman, 2023). CD28, an important co-stimulatory molecule for T cell activation, when expressed at reduced levels, leads to a decline in T cell activation capacity and promotes the onset of immune tolerance (Ye et al., 2023). With aging, T cell subsets often exhibit downregulation or loss of CD28 expression due to repeated and chronic antigenic stimulation (Marija et al., 2022). CD4^+^CD28^−^T cells, as a phenotype of immunosenescence, possess strong pro-inflammatory cytokine secretion (Luis et al., 2022). and perforin/granzyme-mediated cytotoxic capacity (Hammer et al., 2021), promoting and sustaining chronic inflammation. Notably, in this study, lower peripheral absolute counts of CD4^+^CD28^−^ T cells were independently associated with increased CRF risk. This counterintuitive finding may reflect late-stage immune exhaustion induced by chronic tumor- or therapy-related antigenic stimulation, resulting in depletion from the peripheral circulation, while these cells may persist in inflamed tissues and maintain pathogenic activity. Additionally, CD38 is a transmembrane protein expressed on T cells (Pérez-Lara et al., 2021), and its high expression is closely associated with immunosenescence (Camacho-Pereira et al., 2016). Studies have demonstrated a positive correlation between CD38 expression levels and various inflammatory factors, including TNF-α, IL-6, and IL-17. This suggests that the persistent inflammation resulting from the accumulation of aging immune cells (Dang et al., 2023) may be a significant driving factor in the development of CRF. Additionally, CD38 expression has been linked to the decline of NAD and the inflammatory response (Zeidler et al., 2022), further supporting the hypothesis that immunosenescence contributes to the pathogenesis of CRF through inflammatory pathways. In summary, the coexistence of CD4^+^CD28^−^T cell depletion and CD4^+^CD38^−^T cell expansion may represent a maladaptive immune remodeling pattern in CRF, wherein one effector pool collapses due to exhaustion while another expands with abnormal metabolic resilience and inflammatory potential. Together, these changes perpetuate systemic inflammation and fatigue symptoms. Future research should integrate immunophenotyping, metabolic flux analysis, tissue-homing profiling, and longitudinal fatigue assessment to clarify the causal role of these subsets in CRF and evaluate their potential as therapeutic targets.

Inflammation is widely recognized as a key mechanism underlying CRF (García-González et al., 2025). Research indicates that patients experiencing CRF frequently exhibit elevated levels of inflammatory factors such as TNF-α, IL-1β, IL-6, resistin, VEGF-A, and GM-CSF (Schmidt et al., 2024). These inflammatory factors not only play a crucial role in the development and progression of tumors but may also exacerbate fatigue by influencing the nervous system and energy metabolism. In colorectal cancer survivors, impaired energy metabolism resulting from inflammation and mitochondrial dysfunction is considered a potential mechanism contributing to CRF (Van Roekel et al., 2023). Furthermore, chronic inflammation, neuroendocrine dysregulation, and metabolic changes associated with obesity and insulin resistance have been shown to be closely related to the onset of CRF (Zhang and Perry, 2024). Research has additionally found that the gut microbiota of patients with advanced lung CRF correlates significantly with the severity of CRF; severely fatigued patients display a predominance of pro-inflammatory microbiota, whereas those with mild CRF show an increase in anti-inflammatory microbiota (Wei et al., 2023).

Additionally, immune-related signaling pathways play a significant role in the development of CRF. Type I interferon signaling can induce the expression of pro-inflammatory genes and is closely associated with an increase in monocytes (Bower et al., 2024). Furthermore, the administration of platinum-based chemotherapy drugs enhances the inflammatory response by elevating IL-8 levels and exacerbating anemia, thereby worsening CRF symptoms (Zhang et al., 2021). Commonly used immune-inflammatory indicators, such as the neutrophil-lymphocyte ratio (García-González et al., 2024) and the systemic immune-inflammation index (Sun et al., 2025), have been positively correlated with the incidence and severity of fatigue in CRF, reflecting the impact of systemic inflammation on CRF. Notably, traditional Chinese medicine YiFeiSanjieWan mitigates CRF symptoms by inhibiting the Stat3/HIF-1α/BNIP3 signaling pathway, which is induced by aberrantly activated tumor-related inflammation. This action reduces inflammatory levels in the tumor microenvironment and alleviates excessive mitochondrial autophagy activation (Wu et al., 2023), providing new insights for targeted interventions.

Despite identifying several important factors influencing CRF and constructing a preliminary predictive model, this study has certain limitations. First, the overall sample size was relatively small, particularly in the validation cohort, which may have reduced the statistical power to detect weaker associations and, to some extent, affected the model’s predictive performance. Second, as a single-center retrospective study, it is subject to the risk of selection bias, and the temporal or causal relationships between predictors and CRF cannot be definitively established; moreover, the study population may not be fully representative of the broader cancer patient population. In addition, the decrease in AUC from the training set (0.725) to the validation set (0.581) suggests a potential risk of overfitting, which may limit the model’s predictive performance in real-world clinical settings. Due to the retrospective and single-center nature of this study, external validation could not be conducted. Future studies should aim to validate the model using independent, multicenter datasets and prospective cohorts to comprehensively assess its robustness and enhance its generalizability. Third, although demographic characteristics, disease-related variables, and T cell subset indicators were included, several other established determinants of CRF—such as anemia, treatment type and intensity, comorbidities, inflammatory cytokine levels, immune checkpoint molecule expression, sleep quality, and psychosocial factors including depression and anxiety—were not comprehensively assessed due to incomplete or unavailable data. The omission of these variables may have introduced potential confounding factors, thereby limiting a full representation of the multifactorial nature of CRF. Fourth, the study primarily focused on immunological parameters, which, although providing novel insights into the potential role of immunosenescence, do not capture the full biopsychosocial complexity of CRF pathogenesis. Finally, the current model is mainly based on clinical and immunological variables and lacks integration with multi-omics data such as metabolomics and transcriptomics, thereby constraining an in-depth elucidation of the underlying mechanisms of CRF. Future prospective multicenter studies with larger and more diverse cohorts, incorporating a broader range of biological, clinical, and psychosocial parameters, are warranted to refine predictive models, enhance their generalizability, and support the development of personalized, mechanism-based interventions for CRF.

## 5 Conclusion

This study identified increased age, reduced absolute counts of CD4^+^CD28^−^ T cells, and elevated absolute counts of CD4^+^CD38^−^ T cells as independent risk factors for CRF in pan-cancer patients, as determined by multivariate logistic regression analysis. These findings suggest that chronic inflammation and immune remodeling associated with immunosenescence may contribute to the pathogenesis of CRF. Recognition of these immune alterations may support the early identification of patients at higher risk for CRF and inform individualized risk assessment and management strategies. Based on these results, we developed a CRF risk prediction model that demonstrated measurable discriminative performance within the study cohort. While its simplicity and use of routinely obtainable clinical and immunological indicators suggest potential for broader application, its robustness and generalizability require further validation in larger, prospective, multicenter studies across diverse cancer types and healthcare settings. Upon such validation, the model could be integrated into clinical workflows to trigger targeted interventions for high-risk patients, such as early fatigue screening and proactive symptom management, ultimately aiming to mitigate CRF and improve patient quality of life.

## Data Availability

The original contributions presented in the study are included in the article/Supplementary Material. Further inquiries can be directed to the corresponding authors.

## Abbreviations

AC, absolute counts; AUC, area under the curve; BC, Breast Cancer; BFI, Brief Fatigue Inventory; CRC, Colorectal Cancer; CRF, cancer-related fatigue; DCA, decision curve analysis; EC, Esophageal Cancer; GC, Gastric Cancer; LC, Lung Cancer; PC, Pancreatic Cancer; PCa, Prostate Cancer; PLC, Primary liver cancer; ROC, receiver operating characteristic; SD, Standard deviation; Tn, naïve T cell; TNF-α, tumor necrosis factor-alpha; Treg, regulatory T cell.
