# Supplementary material for: Immune indicators as predictors of cancer-related fatigue: a risk prediction model in pan-cancer patients
Source: Front Aging. 2025 Sep 11;6:1666116. doi: 10.3389/fragi.2025.1666116 (PMC12461227; doi:10.3389/fragi.2025.1666116)
Supplement: Supplementary file 1 [file Supplementaryfile1.docx]

# SUPPLEMENTARY FIGURES


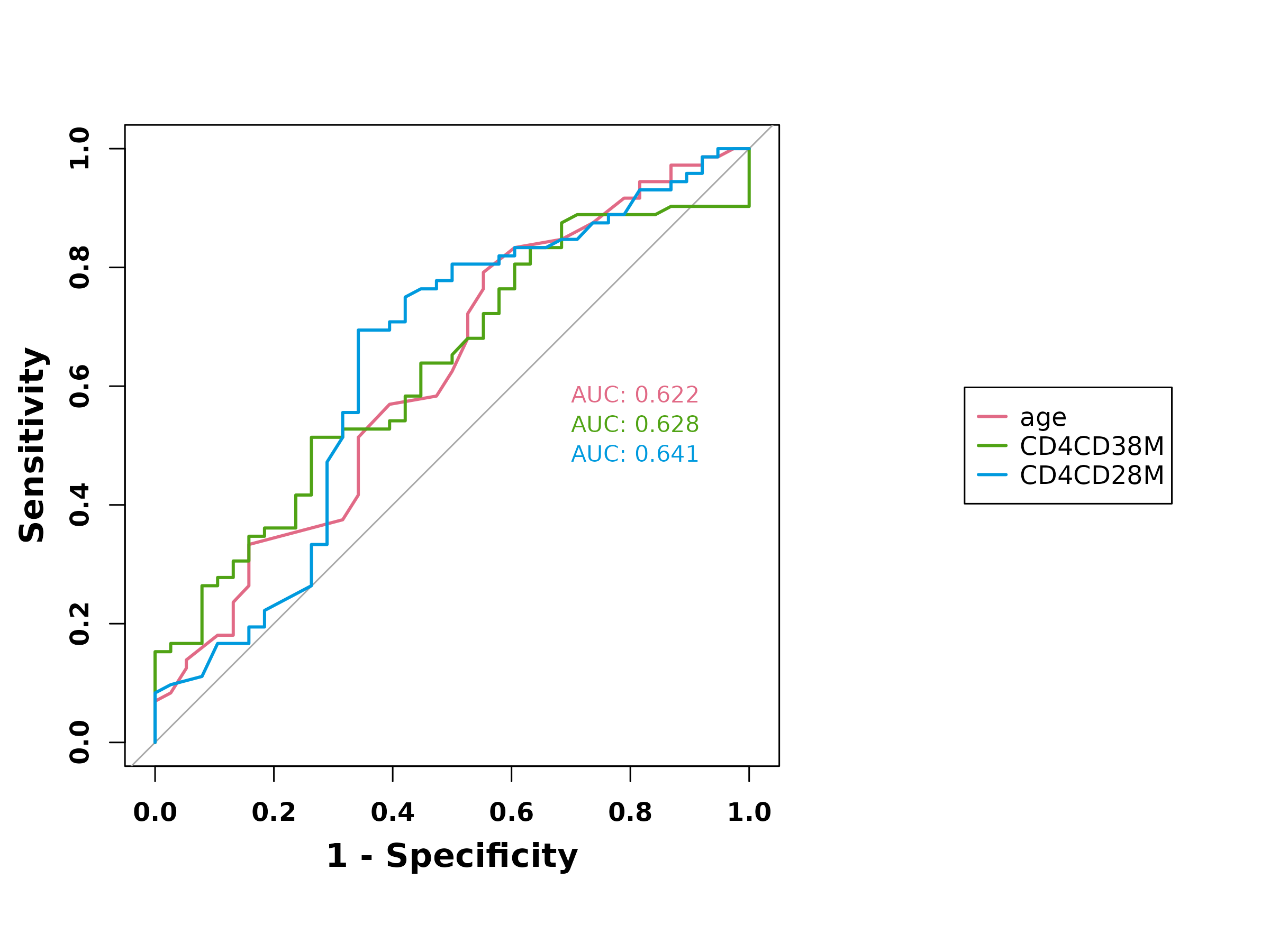


**Fig. S1** ROC curves for three independent predictors of age, CD4CD38M, and CD4CD28M. CD4CD38M, CD4^+^CD38^-^T AC; CD4CD28M, CD4^+^CD28^-^T AC.


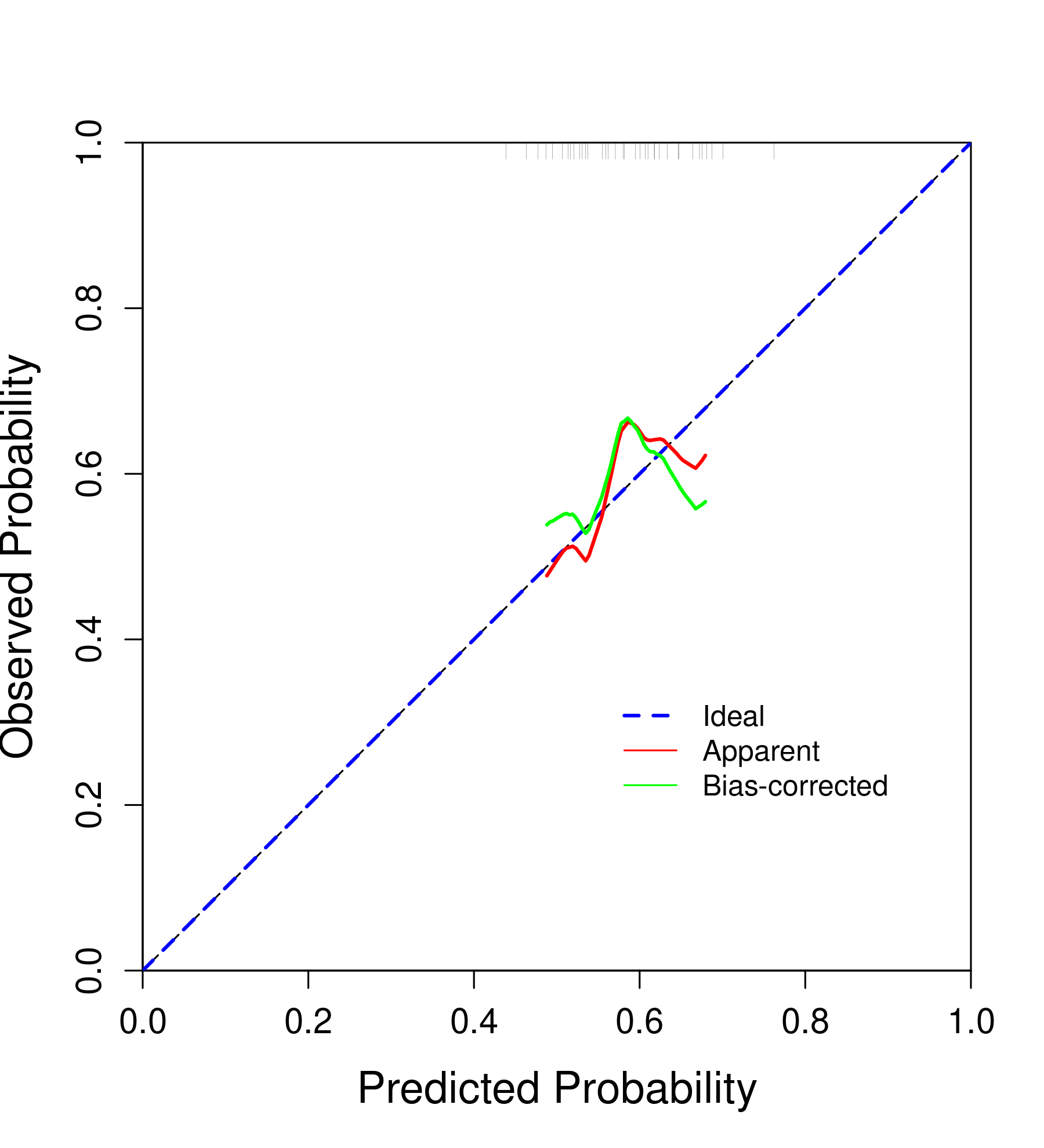


**Fig. S2** Calibration curve of the validation set.


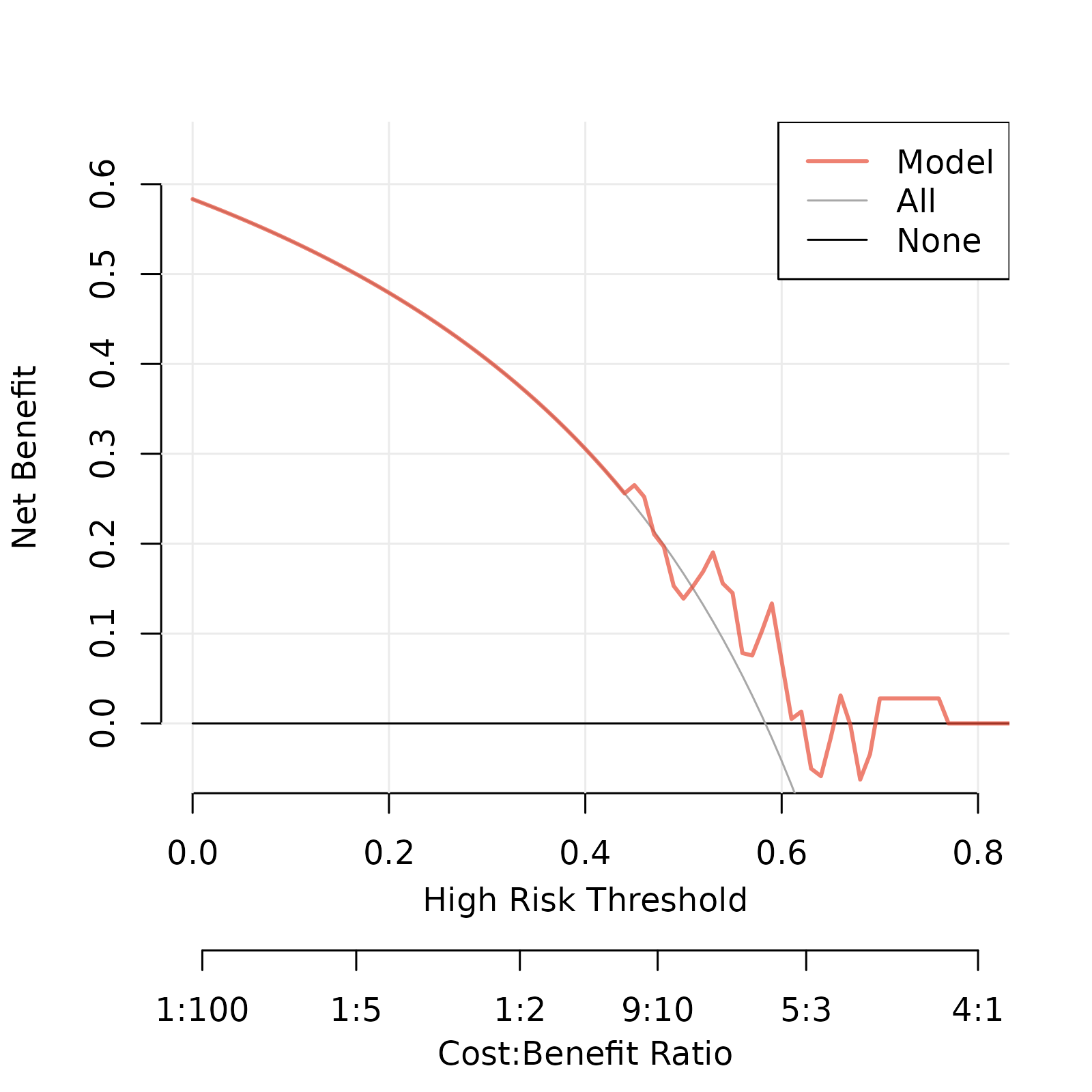


**Fig. S3** DCA curve of validation set.
