# Supplementary material for: Immune indicators as predictors of cancer-related fatigue: a risk prediction model in pan-cancer patients
Source: Front Aging. 2025 Sep 11;6:1666116. doi: 10.3389/fragi.2025.1666116 (PMC12461227; doi:10.3389/fragi.2025.1666116)
Supplement: Supplementary file 2 [file Supplementaryfile2.docx]

# **SUPPLEMENTARY TABLES**

Table S1: Patient demographics and baseline characteristics.

| **Characteristics** | **Training set** | | | **Validation set** | | |
| --- | --- | --- | --- | --- | --- | --- |
|  | **no/mild fatigue** n = 38^1^ | **moderate/severe fatigue** n = 72^1^ | ***p*-value**^2^ | **no/mild fatigue** n = 15^1^ | **moderate/severe fatigue** n = 21^1^ | ***p*-value**^2^ |
| **Age** |  |  | 0.026 |  |  | 0.381 |
| Mean ± SD | 63 ± 11 | 68 ± 9 |  | 64 ± 10 | 61 ± 12 |  |
| **Gender** |  |  | 0.977 |  |  | 0.473 |
| Male | 17 (45%) | 32 (44%) |  | 4 (27%) | 8 (38%) |  |
| Female | 21 (55%) | 40 (56%) |  | 11 (73%) | 13 (62%) |  |
| **Marital status** |  |  | 0.151 |  |  | >0.999 |
| Single | 0 (0%) | 3 (4%) |  | 0 (0%) | 0 (0%) |  |
| Married | 37 (97%) | 60 (83%) |  | 13 (87%) | 18 (86%) |  |
| Divorced | 0 (0%) | 1 (1%) |  | 1 (7%) | 1 (5%) |  |
| Widowed | 1 (3%) | 8 (11%) |  | 1 (7%) | 2 (10%) |  |
| **Clinical stages** |  |  | 0.503 |  |  | 0.296 |
| 0 | 3 (8%) | 4 (6%) |  | 1 (7%) | 2 (10%) |  |
| Ⅰ | 10 (26%) | 13 (18%) |  | 4 (27%) | 1 (5%) |  |
| Ⅱ | 6 (16%) | 12 (17%) |  | 2 (13%) | 1 (5%) |  |
| Ⅲ | 11 (29%) | 17 (24%) |  | 6 (40%) | 11 (52%) |  |
| Ⅳ | 8 (21%) | 26 (36%) |  | 2 (13%) | 6 (29%) |  |
| **Family history** |  |  | 0.641 |  |  | >0.999 |
| No | 31 (82%) | 56 (78%) |  | 13 (87%) | 18 (86%) |  |
| Yes | 7 (18%) | 16 (22%) |  | 2 (13%) | 3 (14%) |  |
| **Smoking history** |  |  | 0.946 |  |  | 0.048 |
| No | 24 (63%) | 45 (63%) |  | 10 (67%) | 7 (33%) |  |
| Yes | 14 (37%) | 27 (38%) |  | 5 (33%) | 14 (67%) |  |
| **Drinking history** |  |  | 0.841 |  |  | 0.200 |
| No | 30 (79%) | 58 (81%) |  | 14 (93%) | 15 (71%) |  |
| Yes | 8 (21%) | 14 (19%) |  | 1 (7%) | 6 (29%) |  |
| **Surgical history** |  |  | 0.834 |  |  | >0.999 |
| No | 24 (63%) | 44 (61%) |  | 10 (67%) | 14 (67%) |  |
| Yes | 14 (37%) | 28 (39%) |  | 5 (33%) | 7 (33%) |  |
| **Radiotherapy** |  |  | 0.483 |  |  | 0.468 |
| No | 29 (76%) | 59 (82%) |  | 12 (80%) | 14 (67%) |  |
| Yes | 9 (24%) | 13 (18%) |  | 3 (20%) | 7 (33%) |  |
| **Chemotherapy** |  |  | 0.365 |  |  | 0.705 |
| No | 14 (37%) | 33 (46%) |  | 3 (20%) | 6 (29%) |  |
| Yes | 24 (63%) | 39 (54%) |  | 12 (80%) | 15 (71%) |  |
| **Immunotherapy** |  |  | 0.126 |  |  | 0.908 |
| No | 25 (66%) | 57 (79%) |  | 9 (60%) | 13 (62%) |  |
| Yes | 13 (34%) | 15 (21%) |  | 6 (40%) | 8 (38%) |  |
| **Targeted therapy** |  |  | 0.824 |  |  | 0.735 |
| No | 23 (61%) | 42 (58%) |  | 8 (53%) | 10 (48%) |  |
| Yes | 15 (39%) | 30 (42%) |  | 7 (47%) | 11 (52%) |  |
| **CD3^+^T AC (cells/μL)** |  |  | 0.916 |  |  | 0.867 |
| Mean ± SD | 676 ± 431 | 668 ± 323 |  | 646 ± 393 | 666 ± 306 |  |
| **CD4^+^T AC (cells/μL)** |  |  | 0.465 |  |  | 0.935 |
| Mean ± SD | 350 ± 160 | 376 ± 201 |  | 327 ± 164 | 332 ± 205 |  |
| **CD8^+^T AC (cells/μL)** |  |  | 0.128 |  |  | 0.651 |
| Mean ± SD | 201 ± 112 | 243 ± 175 |  | 266 ± 238 | 234 ± 154 |  |
| **CD4^+^CD38^+^T AC (cells/μL)** |  |  | 0.016 |  |  | 0.022 |
| Mean ± SD | 34 ± 28 | 54 ± 56 |  | 29 ± 15 | 52 ± 39 |  |
| **CD4^+^CD38^-^T AC (cells/μL)** |  |  | 0.003 |  |  | 0.486 |
| Mean ± SD | 57 ± 33 | 86 ± 67 |  | 83 ± 45 | 73 ± 38 |  |
| **CD4^+^CD28^+^T AC (cells/μL)** |  |  | 0.220 |  |  | 0.946 |
| Mean ± SD | 98 ± 64 | 82 ± 67 |  | 74 ± 42 | 75 ± 33 |  |
| **CD4^+^CD28^-^T AC (cells/μL)** |  |  | 0.024 |  |  | 0.927 |
| Mean ± SD | 16 ± 9 | 13 ± 6 |  | 13.1 ± 5.5 | 13.3 ± 4.8 |  |
| **CD8^+^CD38^+^T AC (cells/μL)** |  |  | 0.742 |  |  | 0.282 |
| Mean ± SD | 42 ± 25 | 40 ± 34 |  | 44 ± 35 | 33 ± 24 |  |
| **CD8^+^CD38^-^T AC (cells/μL)** |  |  | 0.814 |  |  | 0.737 |
| Mean ± SD | 102 ± 50 | 104 ± 52 |  | 103 ± 47 | 99 ± 33 |  |
| **CD8^+^CD28^+^T AC (cells/μL)** |  |  | 0.069 |  |  | 0.672 |
| Mean ± SD | 119 ± 77 | 153 ± 120 |  | 114 ± 58 | 123 ± 67 |  |
| **CD8^+^CD28^-^T AC (cells/μL)** |  |  | 0.135 |  |  | 0.697 |
| Mean ± SD | 20 ± 9 | 23 ± 8 |  | 22 ± 7 | 23 ± 11 |  |
| **Treg AC (cells/μL)** |  |  | 0.081 |  |  | 0.697 |
| Mean ± SD | 32 ± 18 | 26 ± 13 |  | 33 ± 19 | 30 ± 15 |  |
| **CD4^+^Tn AC (cells/μL)** |  |  | 0.700 |  |  | 0.119 |
| Mean ± SD | 16 ± 30 | 14 ± 21 |  | 8 ± 5 | 13 ± 14 |  |
| **CD8^+^Tn AC (cells/μL)** |  |  | 0.573 |  |  | 0.824 |
| Mean ± SD | 8 ± 8 | 7 ± 12 |  | 12 ± 13 | 11 ± 13 |  |
| ^1^n (%) | | | | | | |
| ^2^Welch Two Sample t-test; Pearson's Chi-squared test; Fisher's exact test | | | | | | |

Table S2: The coefficients of Lasso regression analysis.

| Variable | Coefficient |
| --- | --- |
| (Intercept) | 0.4983170738 |
| **Age** | 0.0036392484 |
| **Gender** | 0.0000000000 |
| **Marital status** | 0.0000000000 |
| **Clinical stages** | 0.0000000000 |
| **Family history** | 0.0000000000 |
| **Smoking history** | 0.0000000000 |
| **Drinking history** | 0.0000000000 |
| **Surgical history** | 0.0000000000 |
| **Radiotherapy** | 0.0000000000 |
| **Chemotherapy** | 0.0000000000 |
| **Immunotherapy** | 0.0000000000 |
| **Targeted therapy** | 0.0000000000 |
| **CD3^+^T AC** | 0.0000000000 |
| **CD4^+^T AC** | 0.0000000000 |
| **CD8^+^T AC** | 0.0000000000 |
| **CD4^+^CD38^+^T AC** | 0.0000000000 |
| **CD4^+^CD38^-^T AC** | 0.0006250809 |
| **CD4^+^CD28^+^T AC** | 0.0000000000 |
| **CD4^+^CD28^-^T AC** | -0.0104029720 |
| **CD8^+^CD38^+^T AC** | 0.0000000000 |
| **CD8^+^CD38^-^T AC** | 0.0000000000 |
| **CD8^+^CD28^+^T AC** | 0.0000000000 |
| **CD8^+^CD28^-^T AC** | 0.0000000000 |
| **Treg** | 0.0000000000 |
| **CD4^+^Tn** | 0.0000000000 |
| **CD8^+^Tn** | 0.0000000000 |
